# Rituximab vs. cyclophosphamide in treatment-naive IMN: efficacy and renal protection in early renal impairment

**DOI:** 10.1007/s10157-026-02895-w

**Published:** 2026-06-08

**Authors:** Qi Yu, Qin Yang, Shan Wu, Wenjing Wang, Yangyang Zhang, Yan Liu, Yanlang Yang

**Affiliations:** 1 Department of Nephrology, The First Affiliated Hospital of Wannan Medical University, Yijishan Hospital, Wuhu, 241000 China; 2 Nanchang University, Nanchang, 330031 China

**Keywords:** Membranous nephropathy, Rituximab, Cyclophosphamide, Glomerular filtration rate, Renal function impairment

## Abstract

**Background:**

Cyclophosphamide (CTX) combined with glucocorticoids and rituximab (RTX) are widely used for idiopathic membranous nephropathy (IMN), but clinical data comparing them in newly diagnosed patients with impaired renal function (eGFR < 60 mL/min/1.73 m^2^) remain scarce. This study aimed to compare their efficacy, safety, and effects on renal function in this specific population.

**Methods:**

A retrospective study was conducted at the First Affiliated Hospital of Wannan Medical University from November 2019 to July 2024, including 113 newly diagnosed IMN patients (55 in the CTX group, 58 in the RTX group) diagnosed by renal biopsy or positive PLA2R antibodies. All patients were treatment-naive and followed for ≥ 6 months.

**Results:**

At 6 months, composite remission rates were 69.09% in the CTX group and 62.07% in the RTX group (*P* > 0.05). At 12 months, the RTX group showed a numerically higher composite remission rate (83.87% vs. 70.83% in CTX group) without statistical significance (*P* = 0.186). By 18 months, both groups reached 73.68% composite remission (*P* = 1.000). In the subgroup with eGFR < 60 mL/min/1.73 m^2^, RTX significantly improved renal function at 6 months (*P* = 0.008), while CTX showed no significant improvement (*P* = 0.113); however, no intergroup difference in eGFR was observed (*P* = 0.793). The CTX group had a higher incidence of non-serious adverse events, including leukopenia and mild infections (*P* < 0.05). HypoIgGemia was documented in 5 patients (8.62%) in the RTX group, all mild and asymptomatic.

**Conclusion:**

RTX shows comparable efficacy and better safety relative to CTX in this cohort, and may be a preferred option for newly diagnosed untreated IMN, especially in those with early renal impairment. RTX demonstrated comparable efficacy to CTX in newly diagnosed IMN but with superior safety. Notably, RTX showed greater short-term (6-month) renal function improvement in patients with early renal impairment, though long-term effects need further verification in larger-sample prospective studies.

## Background

Idiopathic membranous nephropathy (IMN) is an autoimmune disease and the most common cause of nephrotic syndrome in adults [1]. It is the second most common glomerular disease in terms of the risk of progression to end-stage renal disease (ESRD) [2]. Long-term follow-up studies have shown that therapy-naive IMN is associated with a high risk of renal function deterioration; approximately 40–50% of therapy-naive patients with persistent nephrotic syndrome (NS) will develop end-stage kidney disease (ESKD) [3, 4]. Therefore, timely clinical intervention and treatment are necessary.

Cyclophosphamide (CTX) combined with glucocorticoid regimens has been proven effective in treating IMN [3, 5]. Although CTX increases the complete remission rate, it also raises the risks of diabetes mellitus, bone marrow toxicity, infection, gonadotoxicity, and cancer [6, 7].

Rituximab (RTX) is a type I chimeric human-mouse anti-CD20 monoclonal antibody, which is expressed on all B cells except precursor B cells and differentiated plasma cells [8]. By binding to the CD20 protein, RTX triggers cell-mediated and complement-mediated cytotoxicity, leading to B cell depletion. As a regimen for refractory IMN, RTX has gained widespread recognition and demonstrated good safety [9, 10].

This study focuses on analyzing the efficacy and safety of CTX combined with glucocorticoids and RTX in the treatment of newly diagnosed IMN, with particular attention to the improvement of renal function in patients with eGFR < 60 mL/min/1.73 m^2^ **(**defined as renal function impairment).

## Methods

### Study population

Patients with IMN from the Department of Nephrology at the First Affiliated Hospital of Wannan Medical University, treated between November 2019 and July 2024, were included.

Inclusion criteria:

1. Diagnosis of IMN by renal biopsy with typical changes, or anti-PLA2R antibody titer > 20 RU/mL in clinically consistent cases;
2. Receipt of RTX or CTX combined with glucocorticoids as initial therapy;
3. Age ≥ 18 years, with complete demographic data, medical history, and laboratory data;
4. Signed informed consent forms.

Exclusion criteria:

1. Follow-up time < 6 months;
2. Secondary MN;
3. Previous use of other immunosuppressive agents;
4. Neoplasms, autoimmune diseases, hematological diseases, or psychiatric disorders;
5. Incomplete clinical or biochemical data;
6. Severe hepatic insufficiency;
7. Pregnancy or breastfeeding.

The research has been approved by the Ethics Committee of the First Affiliated Hospital of Wannan Medical University (approval number: 2020–35). All patients provided written informed consent prior to their inclusion in the study, as evidenced by signed informed consent forms.

Two medication regimens were employed:

- *Cyclophosphamide combined with glucocorticoid group*: The initial oral corticosteroid dose was 1 mg/kg/day (maximum dose 60 mg), which was gradually tapered over 8–12 weeks until discontinuation. Intermittent intravenous CTX was administered monthly at a dose of 10–15 mg/kg for 6 months (usually 0.6–1.0 g/month), followed by additional injections approximately every 3 months over the next 6 months. The total dose of CTX typically reached 6–8 g, not exceeding 10 g. All patients received standard RAS inhibitor treatment as baseline renoprotective therapy unless contraindicated.
- *Rituximab group*: Intravenous injections at a dose of 375 mg/m^2^ once a week for 4 consecutive weeks, or 1 g every 2 weeks (2 doses total as one course). To minimize infusion reactions, subjects received 40 mg of intravenous Methylprednisolone [11] prior to the infusion. At 6 months, anti-PLA2R antibody levels were tested; if the antibodies turned negative, RTX was discontinued. If antibody levels remained unchanged or decreased to < 50 RU/mL but were still present, RTX treatment was continued. All patients received standard RAS inhibitor treatment as baseline renoprotective therapy unless contraindicated.

*Note on RTX dosage*: Among 58 patients in the RTX group, 42 (72.4%) received the standard course (375 mg/m^2^ × 4 weeks), and 16 (27.6%) received 1 g × 2 doses. At 6 months, 28 patients (48.3%) received a second course due to persistent anti-PLA2R antibodies.

### Study outcomes

Primary and secondary outcomes were defined as follows. Primary outcomes: Composite remission rate (complete remission and partial remission) at 6 months.Secondary outcomes: (1) Composite remission rates at 12 and 18 months; (2) Changes in eGFR, 24-h proteinuria, and serum albumin from baseline to 6, 12, and 18 months; (3) Incidence of adverse events during follow-up.

### Treatment response

Treatment response [12, 13] was evaluated based on the following criteria:

- Complete remission (CR): Proteinuria ≤ 0.3 g/24 h, albumin ≥ 35 g/L, and stable renal function.
- Partial remission (PR): Proteinuria of 0.3–3.5 g/24 h or a reduction of > 50% from baseline, with stable renal function.
- No remission: A decrease in 24-h proteinuria of < 50% from baseline or failure to meet the criteria for CR or PR.
- Relapse: Proteinuria exceeding 3.5 g/24 h after remission or an increase of over 50% from the lowest value achieved during PR or CR.
- Composite remission: Either CR or PR.

### Adverse event assessment

Adverse events (AEs) were evaluated and classified according to the Common Terminology Criteria for Adverse Events (CTCAE) version 5.0. Severe adverse events (SAEs) were defined as events that resulted in death, life-threatening conditions, hospitalization or prolonged hospitalization, persistent significant disability/incapacity, or congenital anomalies/birth defects. Non-serious adverse events were thoses that did not meet the criteria for SAEs but required medical intervention or caused discomfort to the patients. AEs were systematically collected during regularfollow-yp visits, and their relationship to the study drugs was assessed by attending physicians.

### Calculation of glomerular filtration rate

The glomerular filtration rate (GFR) was calculated using the Chronic Kidney Disease Epidemiology Collaboration (CKD-EPI) formula: 141 × min (Scr/κ, 1)α × max (Scr/κ, 1) – 1.209 × 0.993^age × 1.018 (if female), where Scr is serum creatinine (mg/dL), κ is 0.7 for females and 0.9 for males, α is –0.329 for females and –0.411 for males, min represents the minimum of Scr/κ or 1, and max represents the maximum of Scr/κ or 1 [14, 15].

### Statistical analysis

Statistical analyses were performed using SPSS 26.0 and GraphPad Prism 8.0 software. The Shapiro–Wilk test was used to assess normality. Continuous variables following a normal or approximately normal distribution were expressed as mean ± standard deviation (SD). Non-normally distributed data were presented as median with interquartile range (IQR). Qualitative (frequency) data were counted with percentages, and differences were assessed using the chi-square test. All probabilities were two-sided, and *P* < 0.05 was considered statistically significant.

## Results

### Baseline characteristics

**Fig. 1:**
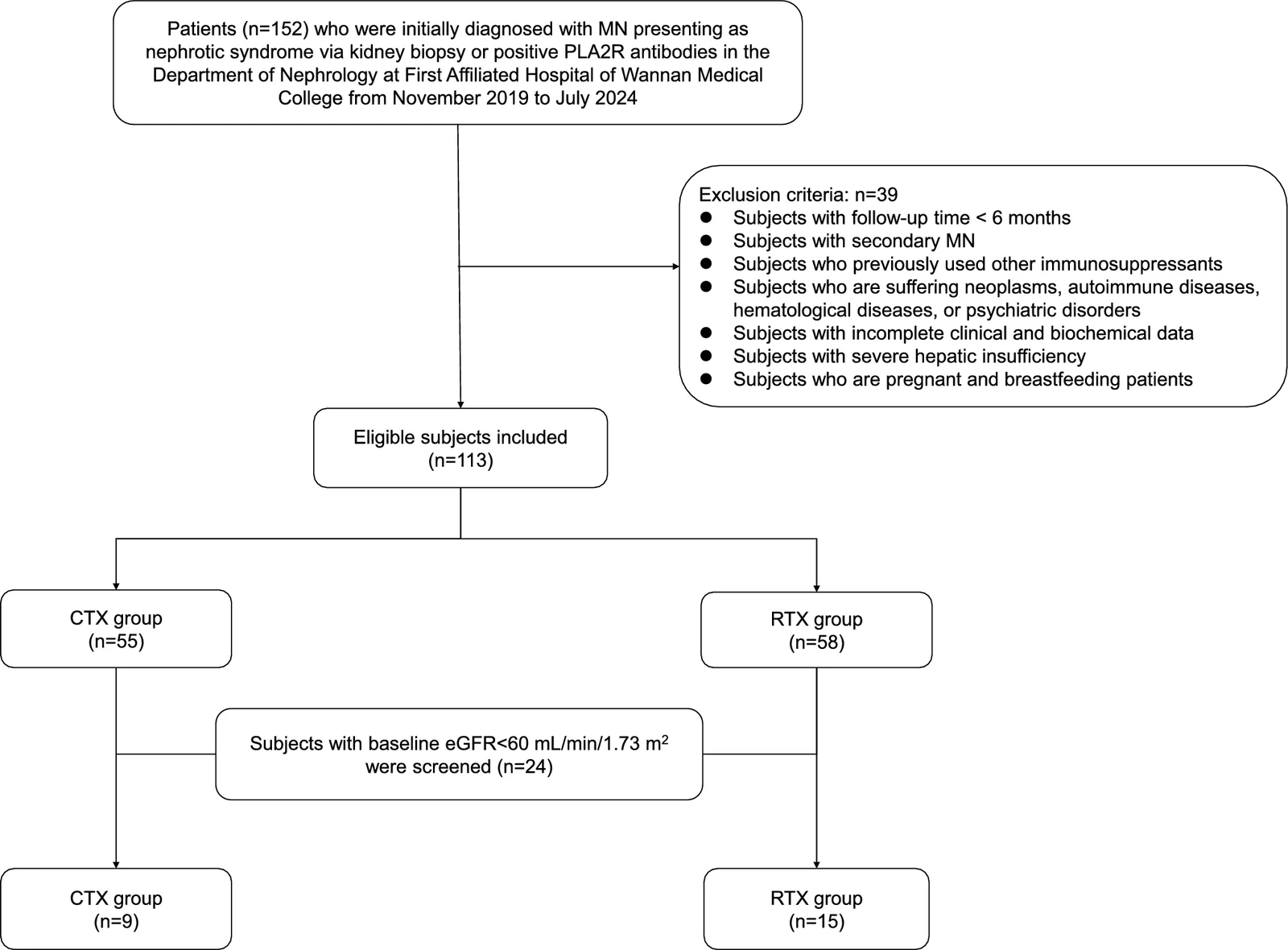
Flowchart of patient enrollment and grouping. IMN, idiopathic membranous nephropathy; CTX, cyclophosphamide; RTX, rituximab; eGFR, estimated glomerular filtration rate.

A total of 152 subjects were initially screened (Fig. 1). Following inclusion and exclusion criteria, 113 IMN subjects were included: 55 in the cyclophosphamide combined with glucocorticoid group and 58 in the rituximab group. Of the 113 patients, 87 (77.0%) were diagnosed by renal biopsy with typical pathological features of IMN, and 26 (23.0%) by high anti-PLA2R antibody (> 20 RU/mL) with consistent clinical manifestations. There was no significant difference in diagnostic composition between groups (*P* = 0.63). Of the 113 patients, 24 (21.2%) had impaired renal function (eGFR < 60 mL/min/1.73 m^2^): 9 in the cyclophosphamide combined with glucocorticoid group and 15 in the rituximab group. Anti-PLA2R antibody positivity rate was 21.8% (12/55) in the CTX group and 24.1% (14/58) in the RTX group, with no significant between-group difference (*P* = 0.76). Compared with the cyclophosphamide combined with glucocorticoid group, the rituximab group had lower high-density lipoprotein levels (1.80 ± 0.44 vs. 1.40 ± 0.30) and higher fasting blood glucose (4.54 ± 0.39 vs. 5.10 ± 0.50) (Table 1).

**Table 1:** Baseline characteristics according to treatment group

Covariates	CTX (n = 55)	RTX (n = 58)	*P*-value
*Demographics*			
Age (years)	55.07 ± 10.93	56.66 ± 14.41	0.56
Gender, Male/Female	36/19	41/17	
Baseline Clinical Data			
Body height (cm)	167.46 ± 6.16	166.50 ± 7.40	0.48
Weight (kg)	67.56 ± 11.11	68.25 ± 12.22	0.69
BMI(kg/m^2^)	24.39 ± 3.22	24.48 ± 3.51	0.75
Blood pressure systolic (mmHg)	133.80 ± 29.82	142.82 ± 33.72	0.36
Blood pressure diastolic (mmHg)	82.11 ± 13.74	82.48 ± 11.17	0.94
*Baseline Laboratory Data*			
Haemoglobin (g/L)	125.84 ± 20.46	123.04 ± 22.37	0.32
24 h urine protein (g/24 h)	4.11(0.26,13.13)	3.58(0.10,15.27)	0.11
Total protein (g/L)	45.67 ± 7.44	48.20(33.80,74.70)	0.14
Albumin (g/L)	23.62 ± 4.57	30.22 ± 8.75	0.06
Globulin (g/L)	22.77 ± 3.39	22.74 ± 4.16	0.95
Blood urea nitrogen (mmol/L)	6.03(2.80,19.07)	5.44(2.68,25.88)	0.21
Creatinine (μmol/L)	74.00(47.90,300.10)	81.25(40.90,389.4)	0.06
eGFR(mLmin/1.73m^2^)	92.97(19.38,128.19)	85.97(15.39,138.27)	0.11
Total cholesterol (mmol/L)	7.22 ± 1.83	6.79 ± 2.10	0.21
Triglycerides (mmol/L)	2.07(0.61,11.04)	1.86(0.67,14.30)	0.29
High density lipoprotein (mmol/L)	1.80 ± 0.44	1.40 ± 0.30	**0.00**
Low density lipoprotein (mmol/L)	4.61 ± 1.43	2.78 ± 1.11	0.28
Glucose (mmol/L)	4.54 ± 0.39	5.10 ± 0.50	**0.01**
Total T cells (%)	78.86 ± 10.63	76.22 ± 10.73	0.47
B lymphocytes (%)	11.95 ± 6.12	9.80 ± 7.56	0.38
Anti-PLA2R antibody titer (RU/mL)	117.05(0.00,874.78)	133.37(0.00,924.76)	0.95
Anti-PLA2R antibody positivity, n(%)	12 (21.8%)	14(24.1%)	0.76
Absolute CD8^+^ T cell count (cells/μL)	363.50 ± 237.46	371.00 ± 165.82	0.78
Absolute CD4^+^ T cell count (cells/μL)	676.25 ± 444.87	864.15 ± 317.47	0.94
Absolute B cell count (cells/μL)	233.13 ± 169.04	220.14 ± 182.35	0.86
Absolute T cell count (cells/μL)	1041.75 ± 664.92	1314.31 ± 502.78	0.86
RAS inhibitor user, n(%)	55(100%)	58(100%)	1.00

For continuous variables, data are expressed as mean ± SD or median IQR, and for categorical variables, data are expressed as n (%). eGFR: estimated glomerular filtration rate; BMI: body mass index;  Note: Bold values indicate statistically significant differences between groups (*P*<0.05)

### Primary outcomes

At 6 months, 38/55 (69.09%) in the cyclophosphamide combined with glucocorticoid group and 36/58 (62.07%) in the rituximab group achieved composite remission (*P* = 0.433). At 12 months, composite remission rates were 70.83% (34/48) vs. 83.87% (26/31) (*P* = 0.186). At 18 months, both groups had a composite remission rate of 73.68% (28/38 vs. 14/19, *P* = 1.000) (Table 2).

**Table 2:** Complete and composite remission at 6–18 months

Follow-up Endpoint	CTX	RTX	χ^2^	*P*-value
CR				
6 months	8/55(14.55%)	6/58(10.34%)	0.459	0.498
12 months	14/48(29.17%)	8/31(25.81%)	0.106	0.745
18 months	13/38(34.21%)	7/19(36.84%)	0.039	0.844
CR or PR				
6 months	38/55(69.09%)	36/58(62.07%)	0.616	0.433
12 months	34/48(70.83%)	26/31(83.87%)	1.753	0.186
18 months	28/38(73.68%)	14/19(73.68%)	0.000	1.000

CR: Complete Remission; PR: Partial Remission; Composite Remission (Complete or Partial Remission)

### Laboratory indicator improvements

**Fig. 2:**
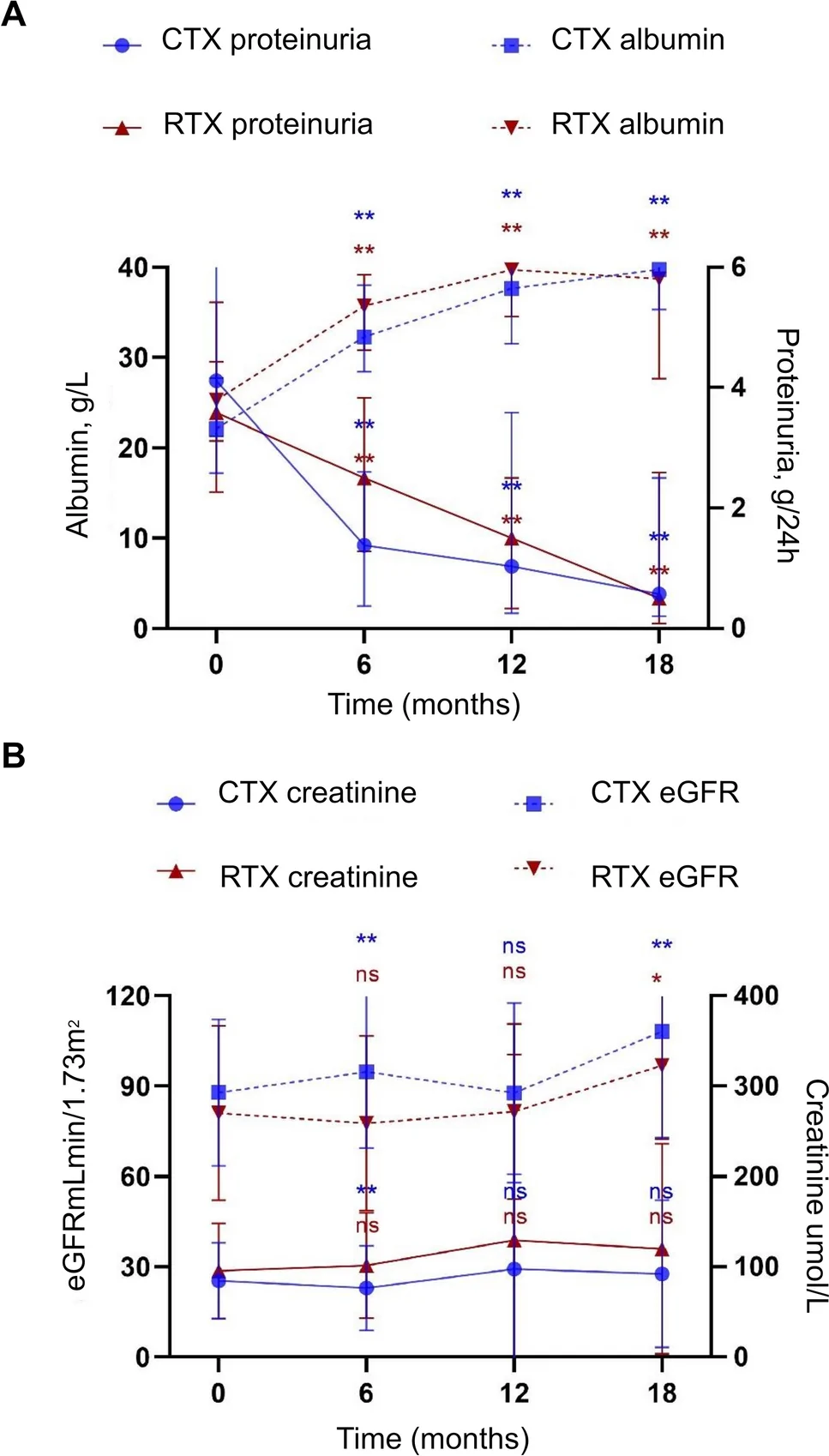
Changes in clinical and renal parameters over 18 months. (A) 24-hour proteinuria and serum albumin levels; (B) Serum creatinine and eGFR levels. CTX, cyclophosphamide; RTX, rituximab; *P<0.05 vs. baseline; **P<0.01vs. baseline; ns, non-significant.

After treatment, 24-h proteinuria decreased and serum albumin increased significantly in both groups from baseline to 18 months (*P* < 0.01) (Fig. 2A).

For renal function:

- Changes in key laboratory parameters at 6 months are summarized in Table 3. In the overall population, cyclophosphamide combined with glucocorticoids improved eGFR at 6 months (*P* < 0.01vs. baseline) and 18 months (*P* < 0.01 vs. baseline), while rituximab showed no significant changes at 6 months and significant increase at 18 months (*P* < 0.05) (Table 3).
- In patients with eGFR < 60 mL/min/1.73 m^2^, rituximab significantly improved eGFR at 6 months (42.13 ± 9.59 vs. 59.39 ± 27.63, *P* = 0.008), while cyclophosphamide combined with glucocorticoids showed no significant improvement (42.23 ± 14.13 vs. 56.25 ± 28.75, *P* = 0.113). There was no significant difference in 6-month eGFR between the two groups (*P* = 0.793) (Table 4).

**Table 3:** Changes in laboratory indicators at 6 months

Laboratory indicators	Treatment group	Before treatment	Post-treatment	*P*-value
24 h Urine protein (g/24 h)	CTX	4.11(0.26,13.13)	1.01(0.01,6.51)	0.00
	RTX	4.58(0.10,15.27)	2.44(0.00,19.45)	0.00
Albumin (g/L)	CTX	23.00 ± 6.75	32.40(11.5,43.5)	0.00
	RTX	25.40(14.30,42.70)	33.00(11.30,45.70)	0.00
Creatinine (μmol/L)	CTX	74.00(47.90,300.10)	65.40(27.90,329.80)	0.00
	RTX	81.25(40.90,389.40)	84.15(33.8,361.40)	0.65
eGFR (mLmin/1.73m^2^)	CTX	92.97(19.38,128.19)	100.70(15.24,134.06)	0.00
	RTX	85.97(15.39,138.27)	81.62(15.92,135.63)	0.68
Total cholesterol (mmol/L)	CTX	7.20 ± 1.84	3.17(3.63,12.54)	0.00
	RTX	6.12(3.02,13.12)	4.99(3.10,10.74)	0.00
Triglycerides (mmol/L)	CTX	2.07(0.61,11.04)	2.16(0.53,11.27)	0.85
	RTX	1.81(0.67,14.30)	1.63(0.63,8.12)	0.00
High density lipoprotein (mmol/L)	CTX	1.81 ± 0.44	1.92 ± 0.43	0.06
	RTX	1.47(0.85,2.74)	1.27(0.73,2.71)	0.00
Low density lipoprotein (mmol/L)	CTX	4.20(1.93,7.21)	2.95(1.57,6.48)	0.00
	RTX	3.69(1.54,9.55)	3.10(1.69,7.64)	0.00

**Table 4:** Improvement in patients with abnormal eGFR

	CTX (n = 9)	RTX (n = 15)	*P*-value
Baseline eGFR	42.23 ± 14.13	42.13 ± 9.59	0.726
eGFR at 6 months	56.25 ± 28.75	59.39 ± 27.63	0.793
P-value	0.113	0.008	

Subjects with eGFR < 60 mL/min/1.73m^2^ were selected for a 6-month follow-up

### Adverse events

A total of 47 subjects experienced adverse reactions: 26 (47%) in the cyclophosphamide combined with glucocorticoid group and 21 (36%) in the rituximab group (*P* = 0.093). The cyclophosphamide combined with glucocorticoid group had a higher incidence of non-serious adverse events (27% vs. 21%, *P* = 0.039), other infections (4% vs. 0%, *P* = 0.046), and leukopenia (11% vs. 2%, *P* = 0.003) (Table 5). In the RTX group, 5 patients (8.62%) developed mild hypoIgGemia, which was asymptomatic and did not require intravenous immunoglobulin supplementation. No fatal adverse events occurred.

**Table 5:** Adverse events according to treatment group

Events	CTX (n = 55)		RTX (n = 58)		*P*-value
	Subjects (%)	Events (%)	Subjects (%)	Events (%)	
All Adverse Events	26(47)	57(100)	21(36)	45(100)	0.093
Serious Adverse Events	11(20)	11(19)	11(19)	11(24)	1.000
Fatal	0(0)	0(0)	0(0)	0(0)	1.000
Non-fatal	10(18)	10(18)	11(19)	11(24)	0.758
Non-Serious Adverse Events	15(27)	46(80)	13(21)	33(73)	**0.039**
Adverse Event					
Pyrexia	12(22)	15(26)	11(19)	12(27)	0.414
Pulmonary Infection	7(13)	7(12)	9(16)	9(20)	0.480
Other infections	2(4)	2(4)	0(0)	0(0)	**0.046**
Hyperglycemia	6(11)	6(11)	3(5)	3(7)	0.157
Leukopenia	6(11)	7(12)	1(2)	1(2)	**0.003**
Hyperkalaemia	2(4)	2(4)	1(2)	1(2)	0.414
Infusion Reactions	2(4)	2(4)	5(9)	5(11)	0.109
Diarrhoea	2(4)	2(4)	1(2)	1(2)	0.709
Intestinal obstruction	0(0)	0(0)	0(0)	0(0)	1.000
Palpitation	3(5)	3(5)	2(3)	2(4)	0.527
Nausea and vomiting	3(5)	4(7)	3(5)	3(7)	0.593
Dizziness and headache	1(2)	1(2)	2(3)	2(4)	0.414
Pain	2(4)	2(4)	3(5)	3(7)	0.527
Worsening renal function	3(5)	3(5)	2(3)	2(4)	0.527
ERSD	1(2)	1(2)	0(0)	0(0)	0.157
ACS	0(0)	0(0)	0(0)	0(0)	1.000
Neoplasm	0(0)	0(0)	0(0)	0(0)	1.000
hypoIgGemia	4(7.3)	4(7.0)	5(8.6)	5(11.1)	0.732

ACS: acute coronary syndrome; ERSD: end-stage renal disease; Infusion reactions include rash, erythema, pruritus, chest tightness, runny nose, and irritability; Hyperglycemia: initial fasting blood glucose normal, occurrence of fasting blood glucose > 6.1 mmol/L at least twice during follow-up; Pulmonary infection: diagnosed as pulmonary infection by chest CT; Leukopenia: ≤ 4 × 10^9/L

## Discussion

This study compared the efficacy and safety of cyclophosphamide combined with glucocorticoids and rituximab in newly diagnosed IMN patients, with a focus on those with impaired renal function (eGFR < 60 mL/min/1.73 m^2^).

Existing studies on rituximab (RTX) and cyclophosphamide (CTX) in membranous nephropathy (MN) have long faced a key limitation: most cohorts include refractory patients or those with prior immunosuppressive exposure, which may mask the true efficacy of initial therapies. A landmark review by Gauckler et al. [16] summarized that RTX has become a first-line option for moderate-to-high-risk MN, but noted that "data on treatment-naive patients, especially those with impaired renal function, remain scarce." Our study addresses this gap by focusing exclusively on newly diagnosed, untreated idiopathic membranous nephropathy (IMN) patients, providing the following novel insights.

RTX shows superior short-term renal protection in newly diagnosed patients with impaired renal function. Gauckler et al. pointed out that progressive renal function decline (eGFR < 30 mL/min/1.73 m^2^) is associated with reduced treatment response, and patients with advanced chronic kidney disease (CKD) are rarely included in clinical trials [16]. Existing data on RTX in this subgroup are limited to small retrospective studies: Hanset et al. reported that 9 of 13 RTX-treated patients with CKD stage 4–5 achieved remission, but the cohort included mixed populations with prior treatments [17].

In contrast, our study focused on newly diagnosed patients with eGFR < 60 mL/min/1.73 m^2^ (a more reversible stage of renal impairment) and found that RTX significantly improved eGFR at 6 months (*P* = 0.008), while CTX combined with glucocorticoids showed no significant improvement (*P* = 0.113). This difference may stem from the "treatment-naive" nature of our cohort: newly diagnosed patients have less glomerular scarring and preserved podocyte function, allowing RTX to exert its B-cell depletion effect more effectively—rapidly reducing PLA2R antibody titers and halting immune-mediated glomerular damage [6, 16]. In contrast, CTX, which acts more slowly and may have cumulative nephrotoxicity [18], showed a weaker renal protective effect in this early-stage impaired subgroup. This finding complements the KDIGO guideline’s recommendation that RTX is preferred for moderate-to-high-risk patients [19], and further specifies that RTX may be more advantageous in newly diagnosed patients with early renal impairment—a population not systematically studied in previous trials.

Anti-PLA2R antibody status was balanced between groups and did not significantly influence remission rates in this study.This support that both regimens are effective across PLA2R-positive and clinically diagnosed IMN in routine practice.

Previous studies, including van den Brand et al., demonstrated that RTX has fewer serious adverse events (SAEs) than CTX, especially in reducing infection and malignancy risks [20]. Gauckler et al. further noted that RTX avoids the gonadotoxicity and urotoxicity of alkylating agents, making it safer for long-term use [16]. However, these studies rarely distinguished between "treatment-naive" and “refractory” patients—refractory patients often have more comorbidities, which may confound safety assessments.

Our study focused on treatment-naive patients and found that RTX not only reduced severe infections (consistent with prior data) but also had fewer non-serious adverse events (21 vs. 27%, *P* = 0.039), particularly in reducing “mild infections” (e.g., urinary tract infections) and leukopenia (2% vs. 11%, *P* = 0.003). This is clinically meaningful: for newly diagnosed patients requiring long-term follow-up, non-serious events are more likely to affect treatment adherence. CTX’s higher incidence of leukopenia (11%) may be related to its bone marrow toxicity [6], while RTX’s adverse events were mainly mild infusion reactions (9%), which are manageable with premedication [16].

Notably, our finding that "RTX reduces non-serious infections" complements van den Brand et al.’s conclusion that RTX has fewer SAEs [20]. By excluding patients with prior treatment exposure, we more accurately reflect the intrinsic safety profiles of RTX and CTX—providing strong evidence for choosing RTX as initial therapy.

Our study holds distinct innovative value compared to previous research. Unlike most prior studies that included mixed populations with refractory cases or those who had received prior treatments [16, 21–23], our research exclusively focuses on newly diagnosed, treatment—naive IMN patients. This eliminates the interference of previous therapies and more authentically reflects the initial efficacy of the treatments. In terms of subgroup analysis, while existing studies mostly involved small samples of patients with advanced chronic kidney disease (eGFR < 30 mL/min) [17], we targeted patients with early renal impairment (eGFR 30–60 mL/min) and were the first to confirm that rituximab has a short—term renal protective effect in this reversible stage. Regarding treatment strategies, previous studies often adopted fixed courses of rituximab or cyclophosphamide, or adjusted retreatment based on clinical responses [16, 19, 21, 22], whereas our study validated that PLA2R antibody—guided rituximab retreatment in treatment—naive patients can achieve a higher remission rate. In terms of safety analysis, unlike previous studies that mainly focused on serious adverse events such as severe infections and malignancies [16] we found that rituximab reduces the incidence of non—serious events (such as mild infections), which is crucial for maintaining long—term treatment adherence in newly diagnosed patients.

The main limitation of this study is the relatively small sample size (113 cases) and single-center retrospective design. Conclusions regarding first-line preference should be interpreted with caution and validated in larger prospective randomized controlled trials. Future studies should: (1) Extend follow-up to assess long-term renal outcomes (e.g., 5-year ESRD risk), especially in the renal impairment subgroup; (2) Explore the mechanism of RTX’s renal protection in newly diagnosed patients (e.g., changes in podocyte markers or urinary IgG levels [17].

In summary, by focusing on treatment-naive newly diagnosed IMN patients, our study not only confirms RTX’s comparable efficacy and superior safety to CTX (consistent with existing guidelines [16, 19]), but also innovatively clarifies its advantages in early renal impairment subgroups and personalized retreatment strategies. These findings support RTX as a favorable initial treatment option, but conclusions should be tempered by the single-center and retrospective design.These findings provide more targeted evidence for clinical decision-making, bridging the gap between current guidelines and real-world practice in treating newly diagnosed IMN.

## Data Availability

The datasets used during the current study are available from the corresponding author on reasonable request.
